# Beaver Colony Density Trends on the Chequamegon-Nicolet National Forest, 1987 – 2013

**DOI:** 10.1371/journal.pone.0170099

**Published:** 2017-01-12

**Authors:** Christine A. Ribic, Deahn M. Donner, Albert J. Beck, David J. Rugg, Sue Reinecke, Dan Eklund

**Affiliations:** 1 US Geological Survey, Wisconsin Cooperative Wildlife Research Unit, University of Wisconsin, Madison, Wisconsin, United States of America; 2 Institute for Applied Ecosystem Studies, Northern Research Station, US Forest Service, Rhinelander, Wisconsin, United States of America; 3 Wisconsin Cooperative Wildlife Research Unit, Department of Forest and Wildlife Ecology, University of Wisconsin, Madison, Wisconsin, United States of America; 4 Northern Research Station, US Forest Service, Madison, Wisconsin, United States of America; 5 Chequamegon-Nicolet National Forest, US Forest Service, Park Falls, Wisconsin, United States of America; Auburn University, UNITED STATES

## Abstract

The North American beaver (*Castor canadensis*) is a managed species in the United States. In northern Wisconsin, as part of the state-wide beaver management program, the Chequamegon-Nicolet National Forest removes beavers from targeted trout streams on U.S. Forest Service lands. However, the success of this management program has not been evaluated. Targeted removals comprise only 3% of the annual beaver harvest, a level of effort that may not affect the beaver population. We used colony location data along Forest streams from 1987–2013 (Nicolet, northeast Wisconsin) and 1997–2013 (Chequamegon, northwest Wisconsin) to assess trends in beaver colony density on targeted trout streams compared to non-targeted streams. On the Chequamegon, colony density on non-targeted trout and non-trout streams did not change over time, while colony density on targeted trout streams declined and then stabilized. On the Nicolet, beaver colony density decreased on both non-targeted streams and targeted trout streams. However, colony density on targeted trout streams declined faster. The impact of targeted trapping was similar across the two sides of the Forest (60% reduction relative to non-targeted trout streams). Exploratory analyses of weather influences found that very dry conditions and severe winters were associated with transient reductions in beaver colony density on non-targeted streams on both sides of the Forest. Our findings may help land management agencies weigh more finely calibrated beaver control measures against continued large-scale removal programs.

## Introduction

In Wisconsin, as in many states, the North American beaver (*Castor canadensis*) is a managed species. Wisconsin’s management plan addresses a wide variety of issues important to citizens of the state, making the plan a compromise of the stakeholders’ concerns [1]. One objective of the plan since the 1930s [2] has been to protect cold-water trout fisheries (primarily native brook trout). The perception underlying this protection is that beaver dams on cold water streams may cause an increase in stream temperature, which may render some streams unsuitable for native trout [3]. To counter this, Wisconsin’s beaver management plan recommends direct beaver control on trout streams. This protection is primarily implemented in northern Wisconsin, which has an abundance of cold-water streams and relatively high beaver density [1]. The direct control tools used to manage beaver populations in Wisconsin are “regulated trapping” (i.e., permitted trapping by the public for beaver pelts) and “targeted trapping” by government agencies, primarily under direction of Wildlife Services, US Department of Agriculture Animal and Plant Health Inspection Service (USDA-WS). The effectiveness of the beaver management program has not been evaluated, although we do know that regulated trapping of beavers does not always result in reductions of the local beaver population [4]. Moreover, targeted removals comprise only 3% of the annual beaver harvest in Wisconsin [1]. It is not clear, a priori, that this level of effort is sufficient to measurably affect the beaver population of interest.

The Chequamegon-Nicolet National Forest (hereafter “Chequamegon-Nicolet NF” or “Forest”) is the steward of an economically significant cold-water trout fishery in northern Wisconsin. To help maintain that fishery, the Forest has been open to regulated trapping, and has partnered in the state-wide beaver management program for many years [1, 5]. Specifically, the Chequamegon-Nicolet NF has had a targeted removal program since the late 1980s that removes beavers and their dams from selected trout streams. The Forest was interested in an evaluation of their program, as that would provide managers with the necessary information to better evaluate, plan, and implement beaver management programs across large landscapes.

In this paper, we examine the temporal and spatial variation in beaver colony densities across the Chequamegon-Nicolet NF during the targeted beaver management program. The specific objectives are to (1) determine whether the direct control program produced a detectable effect on beaver colony density on targeted streams, (2) if there was an effect, determine whether the effect is substantial enough to conclude that the program is being successful, and (3) explore whether large-scale patterns in temperature or precipitation affected beaver colony density trends on streams without targeted removals.

Weather is known to affect annual variation in beaver populations [6] via multiple pathways. Therefore, the third objective addresses two questions. First, does weather over the period of study explain deviations around a larger time trend, thereby improving ability to detect colony trends? Second, given a changing climate regime [7], does variability in weather serve as a driver of time trends?

Overall, we found that the Chequamegon-Nicolet NF’s targeted management program was able to materially reduce the beaver population on targeted streams. In our exploratory analyses of weather effects, we found that very dry conditions and severe winters explained some annual variability in colony density, but neither were strong large-scale drivers of beaver population trends on non-targeted streams. Our findings may be used to help refine management options across the Forest and the state to lower the cost of successful beaver management.

## Materials and Methods

### Study Area

**Fig 1:**
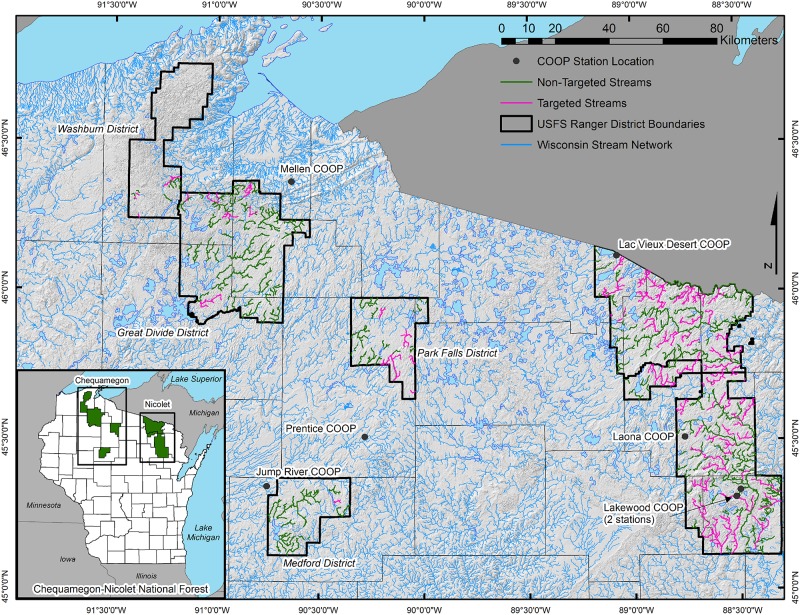
Location of the Chequamegon-Nicolet National Forest and the northern Wisconsin stream network. Streams surveyed during fall for active beaver colonies on the Chequamegon-Nicolet National Forest. Targeted streams are in magenta and non-targeted streams are in green. The grey background for the stream network is the topography of northern Wisconsin as represented by a hillshade model. Weather data were obtained from the indicated National Weather Service Cooperative Observer Program (COOP) weather stations.

The Chequamegon-Nicolet NF is located in northwestern (Chequamegon side) and northeastern (Nicolet side) Wisconsin (Fig 1). The two sides of the Forest were managed separately until 1998 when they were formally merged into a single administrative unit, the Chequamegon-Nicolet NF. Both sides of the Forest are located in the northern highland province of Wisconsin [8]. This province is generally flat to gently rolling with little topographic variation [9] due to hundreds of millions of years of erosion combined with the effects of repeated glaciation cycles [8]. Streams in the Chequamegon-Nicolet NF are considered to be headwater streams, with over 80% of streams on both sides of the Forest being stream order 1 or 2, and predominantly less than 15 m wide. The streams typically occur in old glacial drainages and wetlands with broad valleys having low slopes, and sand or gravel as dominant sediment [9]. Stream gradient averages 8.9 m/km on the Chequamegon side and 4.1 m/km on the Nicolet side; because of these low gradients, streams are easily dammed [9]. Water temperatures are regulated by groundwater inflow and degree of vegetative shading [10]. The Forest’s vegetation is composed of mesic mixed northern hardwoods [black ash (*Fraxinus nigra*), American elm (*Ulmus americana*), red maple (*Acer rubrum*), sugar maple (*Acer saccharum*), basswood (*Tilia americana*), oak (*Quercus* spp.)], conifer [jack pine (*Pinus banksiana*), red pine (*Pinus resinosa*), balsam fir (*Abies balsamea*), eastern white pine (*Pinus strobus*), northern white cedar (*Thuja occidentalis*), white spruce (*Picea glauca*), black spruce (*Picea mariana*), tamarack (*Larix laricina*)], deciduous trees [quaking aspen (*Populus tremuloides*), bigtooth aspen (*P*. *grandidentata*), balsam poplar (*Populus balsamifera*), paper birch (*Betula papyrifera*)], and riparian shrubs [alder (*Alnus* spp.), willow (*Salix* spp.)].

The average annual temperature is about 4.4°C in northern Wisconsin [11]. During more than one-half of the winters, temperatures fall to -40°C or lower; summer temperatures above 32.2°C occur on average two to four days in northern Wisconsin [11]. However, there is a significant warming trend in northwestern Wisconsin [12]. The long-term mean annual precipitation is 12.6 cm in northern Wisconsin [11] but summer precipitation has declined while autumn precipitation has increased [12]. Average seasonal snowfall is 164 cm and the average date of first snowfall in northern Wisconsin is in early November [11]. Many of the streams and lakes in northern Wisconsin are ice-covered from late November to early April [11].

### Beaver Control and Monitoring Program

The Chequamegon-Nicolet NF classifies its streams into “trout” and “non-trout”, based on the Wisconsin Department of Natural Resources (WDNR) categorization of streams into three trout-relevant classes [13]. “Trout” streams are high quality with natural or stocked trout populations (i.e., WDNR Class 1 or 2). “Non-trout” streams correspond to WDNR Class 3 streams (marginal habitat quality with no natural reproduction). As part of the state beaver management plan, the WDNR determined the high-quality trout streams that needed to be protected using beaver removal. The Chequamegon-Nicolet NF determined which of those streams fell within the borders of the Forest and designated trout streams as “targeted” if those were subject to beaver removal and “non-targeted” for streams that were not. If the WDNR-designated trout streams were in wilderness areas (where no management activities are allowed) or ran through private lands in the Forest with no permission to conduct beaver management from the landowner, those trout streams were moved into the “non-targeted” category. Non-trout streams that were tributaries of targeted trout streams were moved into the “targeted” category. Targeted and non-targeted streams are displayed in Fig 1, and the associated lengths for each of the four categories are presented in Table 1.

**Table 1:** Effort for aerial surveys of beaver colonies made October–November along four categories of streams in the Chequamegon-Nicolet National Forest, 1987–2013 (Nicolet side) and 1997–2013 (Chequamegon side). Targeted streams were subject to the beaver control program.

Side of Forest	Stream type	Years	Stream length flown (km)
Nicolet	Targeted Trout	1987–2013	826.0
	Targeted Non-trout	1987–2013	77.1
	Non-targeted Trout	1987–2013	753.0
	Non-targeted Non-trout	1987–2013	285.7
Chequamegon	Targeted Trout	1997–1999	164.8
		2000–2013	167.5
	Targeted Non-trout	1997–2013	4.9
	Non-targeted Trout	1997–1999	243.31
		2000, 2001, 2003–2006, 2009, 2011–2013	259.9
		2002, 2007, 2008, 2010	264.9
	Non-targeted Non-trout	1997–1998	519.5
		1999	562.1
		2000	591.1
		2001–2013	662.1

The beaver management program started ten years later on the Chequamegon side (1997) than the Nicolet side (1987). As noted above, the Forest is open for regulated trapping but it does not collect information on how many beavers are removed. The WDNR estimates the state-wide beaver harvest using voluntary responses to a questionnaire mailed to a sample of licensed trappers [1].

The Forest’s management program had two components. The first component consisted of contracting the removal of all known beaver colonies and lodges from targeted streams in the spring (April to early May) of each year by USDA-WS [14, 15]. Note that beaver control program activities are not subject to IACUC review, as they are land management activities, not research activities. However, control program activities were conducted in accordance with state law and best management practices sponsored by the Association of Fish and Wildlife Agencies as codified in USDA-WS directives [16].

The second component was fall monitoring of beaver locations on targeted and non-targeted streams to assist in locating active beaver colonies on targeted streams for removal the following spring. The Forest contracted with USDA-WS to conduct fall aerial surveys to document the location of active beaver colonies along its land base (Table 1). The fall flights were land management activities, not research activities, and therefore not subject to IACUC review. Aerial surveys for beaver colonies have been found to be an effective method for examining abundance patterns [6, 17].

Aerial fall flights were conducted after leaf-off, usually mid-October–November. Flights were made between 0900 and 1600 (Central Standard Time) at 213–244 m above the surface, flying at 128–161 km/hr. The Nicolet side was flown by the same contract pilot since monitoring began in 1987. The Chequamegon side was flown by a variety of WDNR pilots. Completing both surveys required approximately 40 hours of flight (approximately 24 hours for Nicolet side and 16 hours for Chequamegon side), and had to be completed after leaf-off and before snowfall to adequately observe current beaver activity. Since 2007, flight paths have been tracked using GPS. Because of the short window between leaf-off and first snowfall, flights were conducted on consecutive days if the pilot assessed the weather conditions to be safe for flying. Adjusting the flight schedule for optimal weather conditions to increase observation conditions was not possible because of the short observation window and large land base to cover.

One USDA-WS biologist made all observations on the Chequamegon side since the flights began; on the Nicolet side, there were 4 biologists. On each flight, the biologist documented the location of colonies based on evidence of recent beaver activity. A colony was considered active if there was a lodge with fresh mud or cuttings and/or a food cache with fresh cuttings. Active colonies were recorded along the length of the stream, typically on one pass. However, if there was heavy conifer cover or steep terrain, the pilot would make a second pass to observe the stream from a different vantage point. The pass was conducted such that the stream was on the observer (or right) side of the plane. Active colonies were marked on a map and then digitized from the map into a geographic information system. Accuracy was estimated at ±322 m. Maps of beaver colony locations made from the aerial surveys were made available to the public by request, and the Forest informed the Wisconsin Trappers Association when the maps became available. No information was available about how regulated trappers used those maps to target streams on the Forest (as noted above).

For this study, we used beaver colony count data from 1987–2013 for the Nicolet side and 1997–2013 for the Chequamegon side. We used data for all stream systems flown for ≥5 years. The response variable was colony density (active beaver colony count/km stream flown) by stream category, year, and side of Forest. Because few non-trout streams were targeted (Table 1), we did not use this category in any further analyses.

### Weather Variables

To explore whether weather variability could explain inter-year variation or trends in beaver colony densities on non-targeted streams, we used the beaver literature to determine what variables to consider in the analysis. We were specifically interested in weather variables that affected survival of the 2-year old beavers that annually disperse from their natal dens to establish their own independent colonies. In a population viability analysis, early-age class survival was critical for beaver population persistence, and large variation in early-age class survival rates made the beaver population vulnerable to extinction [18].

A beaver colony consists of a pair of adult beavers and their young. Young stay in the lodge until they are 2 years of age [19], when they disperse in the spring (April–May) [20]. Proximate factors affecting beaver litter size and survival of the young in the den include quantity and quality of available food and severity of winter weather [18, 19]. Quantity and quality of food resources is affected by weather conditions [21, 22]. For the European beaver (*Castor fiber*) in Norway, higher rainfall in the fall prior to the spring when females give birth led to smaller litter sizes [22]. The higher rainfall stressed important food plants by waterlogging their roots, resulting in lowered nutrient content for beavers during the reproductive period [22]. Reduced survival of young in the den (leading to fewer 2-year-olds available for dispersal) was associated with warmer spring temperatures and increased spring-summer precipitation, both factors that can reduce the nutritional value of plants [21, 22]. However, warmer and wetter conditions during spring dispersal would be favorable for the survival of the 2-year-olds as they move away from the den [23].

In northern areas, beavers rely on winter food caches, generally set aside after the first heavy frost [18]. Severely cold winters can prompt early depletion of food caches, leading to physiological stress and reduced survival [24, 25]. This effect can be offset by heavy snowfall, which improves the insulation of the lodge, reducing thermoregulatory energy demands [24, 25]; lighter than average snowfall would have the opposite effect.

Thus, we chose four broad weather variables as affecting female production of young and survival of 2-year old beavers: temperature during late spring (when plants start to grow, i.e., time when green-up occurs), precipitation as reflected in soil moisture during the growing season, temperature during the winter, and winter snowfall. All weather variables were lagged by up to three years to reflect environmental conditions and potential food nutritional value when the young were in the lodge (lag of 0 to 2 years) or for the female before breeding (lag of 3 years). We then defined our measures of these variables to reflect conditions in northern Wisconsin (Table 2). In northern Wisconsin, green-up occurs in May, and the growing season for aspen (*Populus* spp.) extends through September [26]. In northern Wisconsin, the first heavy frost occurred, on average, in late August to early September [11] though there was a shift of 3–12 days later in the 2000s [12]. We defined winter to be December–March because these months represent the coldest months in northern Wisconsin, and the months when the heaviest snowfall is expected. To represent severe winter conditions, we used annual cumulative snowfall and number of days below −17.8°C (0°F).

**Table 2:** Variables used to assess the ability of weather to explain minor variations in the beaver density index on non-managed streams or function as drivers of trends on those streams. Each variable was computed for the current year and the three preceding years. Variables were standardized against their long-term averages (1983–2013).

Variable description	Period of coverage
Mean maximum daily temperature	Green-up: May
Mean monthly Palmer Drought Severity Index (PDSI)	Growing season: May–September
Mean monthly PDSI, May	Green-up: May
Mean monthly PDSI, June and July	Peak growth: June–July
Mean monthly PDSI, August and September	Growth decline: August–September
Cumulative snowfall	Winter: December–March
Number of days below −17.8°C (0°F)	Winter: December–March

We standardized all weather variables by their long-term (1983–2013) means. This standard statistical transformation put extreme events on the same scale (i.e., anomalous conditions will have large negative or positive values, regardless of the variable) and also made variables comparable across the sides of the Forest. We used the 30-year time span so that the overall average conditions were based on data encompassing the most variability in weather conditions against which to judge extreme events (i.e., incorporates the effects of climate change [12]).

All temperature data were downloaded from PRISM [27], imported into ArcMap [28] using a Python for ArcGIS (ArcPy) script, and given a NAD1983 projection if the data did not already have a projection. PRISM data are provided at a 30 m x 30 m scale of resolution so there are multiple 30 m x 30 m cells per district. As part of the ArcPy script, the tool “Zonal Stats as Table” was then run using the Forest’s management districts as zones. Using the mean function in the tool, data from the 30 m x 30 m cells were averaged over the districts by side of the Forest. For the Chequamegon side, we used a weighted average where the weights were the number of miles flown within a district during the fall flights.

For temperature conditions during the month when green-up occurs, we calculated the mean maximum daily temperature for May of each year. To represent precipitation conditions during the growing season each year, we used the Palmer Drought Severity Index (PDSI), a standardized index for how much water is available to plants [29]. The Nicolet side was covered by the PDSI Northeast Division (4703). On the Chequamegon side, the Washburn/Great Divide district was covered by the PDSI Northwest Division (4701) and the Park Falls and Medford districts were covered by the PDSI North Central Division (4702). An overall Chequamegon PDSI was an average of PDSI for Divisions 4701 and 4702 weighted by the number of miles flown during the fall flights for the districts.

We used cumulative snowfall data from National Weather Service Cooperative Observer Program (COOP) weather stations nearest the Chequamegon-Nicolet NF (Fig 1). Monthly snow data were downloaded from the National Climatic Data Center [30]. Cumulative snowfall for the Chequamegon side was an average weighted by the number of miles flown during the fall flights for the district closest to the COOP stations. Cumulative snowfall for the Nicolet side was the average of the data from the COOP stations.

Number of days with an average daily temperature less than −17.8°C was calculated from daily temperatures for December-March from the PRISM website [27]. For each year, number of days with an average daily temperature less than −17.8°C was calculated by month and then summed for December–March.

### Statistical Models

We analyzed time trends by side of the Forest (Chequamegon, Nicolet) and by 3 stream categories (targeted trout, non-targeted trout, non-targeted non-trout). For time trend analyses on non-targeted streams, we used the trout or non-trout classification as a variable in the model to determine differences between trout and non-trout streams. To explore the influence of weather, we focused on non-targeted streams to avoid the confounding effect of beaver management. We added each weather variable to either a time trend or a constant model (if there was no time trend).

We used generalized additive models with a gamma of 1.4 to avoid overfitting [31] for all analyses. For the time trend models, we also used segmented linear models to estimate when changes in slope occurred. We used a Gaussian error structure with no autocorrelation in all models. To compare models, we used Akaike Information Criteria corrected for small sample size (AIC_c_) [32]. We calculated evidence ratios using AICc weights to determine how good the minimum AIC_c_ model was with respect to the other models [32]. We considered models within 2 AIC_c_ units of the minimum AIC_c_ model to be competitive models [32]. We used the Davies test to determine the significance of the segmented linear models. All analyses were done with R version 3.1.2 [33]. Significance was assessed at a P of 0.05 and trends at 0.10.

## Results

### Time Trends

**Fig 2:**
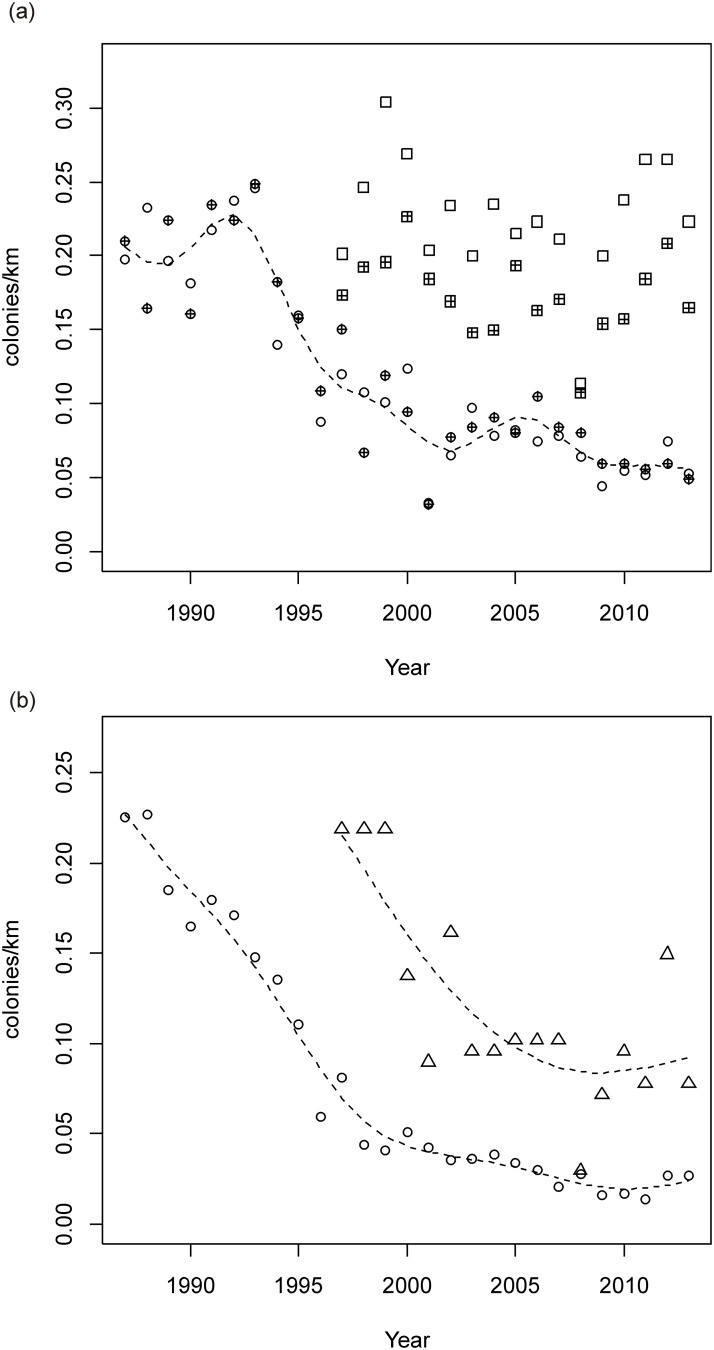
Beaver colony numbers/km by year for (a) trout streams without targeted beaver management and (b) trout streams with targeted beaver management for the Chequamegon-Nicolet National Forest. In panel (a), open circle = Nicolet trout streams, filled circle = Nicolet non-trout streams, open square = Chequamegon trout streams, filled square = Chequamegon non-trout streams. In panel (b), open circle = Nicolet side, triangle = Chequamegon side. Dotted lines are the predicted trends from generalized additive models (significant trends only).

On the Chequamegon side, there was no significant linear trend in beaver colony density on non-targeted streams (Year term: t = −1.1, df = 30, P = 0.29), regardless of whether the stream was a trout or non-trout stream (Trout Classification*Year interaction term, t = 0.169, df = 30, P = 0.87) (Fig 2a). However, there was a difference in colony density between non-targeted trout and non-targeted non-trout streams (Stream Classification term: t = 4.45, df = 30, P < 0.001). Colony density was 0.226 colonies/km (SE = 0.005, n = 17) on non-targeted trout streams compared to 0.173 (SE = 0.005, n = 17) for non-targeted non-trout streams.

On Chequamegon targeted trout streams, there was a significant nonlinear decline in beaver colony density over time (s(Year): F = 11.92, df = 2.1, 23.9, P < 0.001; adjusted R^2^ = 0.663) (Fig 2b). Specifically, there was a change in slope in 2003 (Davies statistic = 2001.5, P < 0.014). In 1997, colony density on targeted trout streams was 0.218 colonies/km, and then declined until 2003 (slope = −0.0219, 95% CI = (−0.0356, −0.0082)). After 2003, colony density stabilized at 0.090 colonies/km (SE = 0.010, n = 10) (slope = 0.0005, 95% CI = (−0.0075, 0.0085)), 60% lower than the colony density found on non-targeted trout streams during that same time period.

On the Nicolet side, beaver colony density on non-targeted streams declined nonlinearly (s(Year): F = 8.1, df = 8.1, 43.9, P < 0.001; adjusted R^2^ = 0.858) (Fig 2a), and there was no difference in colony density between trout and non-trout streams (Stream Classification term: t = −0.38, df = 30, P = 0.71). There were changes in slope in 1993 and 1996 (Davies statistic = 1987, P < 0.0001). From 1987–1993, beaver colony density on non-targeted streams was stable at an average of 0.213 colonies/km (SE = 0.008, n = 14) (slope = 0.006, 95% CI = (−0.001, 0.014)). Colony density declined after 1993 but at a more rapid rate from 1993–1996 (slope = −0.042, 95% CI = (−0.066, −0.019)) compared to 1996–2013 (slope = −0.003, 95% CI = (−0.005, −0.002)). Average beaver colony density on non-targeted streams during the last part of the series (2002–2013) was 0.071 colonies/km (SE = 0.003, n = 24).

Beaver colony density on Nicolet targeted trout streams also declined nonlinearly (s(Year); F = 157.1, df = 5.4, 20.6, P < 0.001; adjusted R^2^ = 0.975) (Fig 2b). There was a change in slope in 1999 (Davies statistic = 2000, P < 0.0001). In 1987, beaver colony density on targeted trout streams was 0.225 colonies/km. From 1987–1999, colony density declined (slope = −0.0161, 95% CI = (−0.0182, −0.0141)). After 1999, colony density continued to decline but at a slower rate (slope = −0.0020, 95% CI = (−0.0035, −0.0005)). Average beaver colony density on targeted trout streams during the last part of the series (2002–2013) was 0.027 colonies/km (SE = 0.002, n = 12), 61% lower than the colony density found on non-targeted streams.

### Exploratory Analysis of Beaver Colony–Weather Relationships on Non-targeted Streams

On the Chequamegon side, when we added weather variables to a model controlling for the differences in beaver colony density on the non-targeted streams, there was a significant positive relationship with June-July PDSI in the year prior to the fall flight (s(June-July PDSI lag 1): F = 9.07, df = 5.3, 27.7, P < 0.001; adjusted R^2^ = 0.77). However, 2008 beaver colony density was an influential point. Specifically, 2008 had the lowest colony density in the series and was linked to the 2007 June-July PDSI, which was the lowest value in the PDSI series and the only one that fell into the PDSI severe drought category. When the 2008 point was removed, the June-July PDSI in the year prior to the fall flight was no longer significant (June-July PDSI lag1: t = −0.00002, df = 30, P = 0.99). When we added an indicator variable for the 2007 drought to the model adjusting for different densities on the non-managed streams, the influence of the severe drought in 2007 was significant (2007 severe drought: t = −4.87, df = 31, P < 0.0001; adjusted R^2^ = 0.627). When we added weather variables to the 2007 drought model, the minimum AIC model linked colony density to May temperature 3 years before the fall flight (i.e., conditions affecting females before pregnancy). A competitive model included severe winter temperatures the year of the fall flight (i.e., winter before dispersal of the 2-year-olds) (Table 3). For the minimum AIC model, there were higher colony densities in year i when there were cooler May temperatures three years prior to the fall flight (coefficient = −0.006; t = −2.88, df = 30, P = 0.007; adjusted R^2^ = 0.698). This model was only 1.6 times better than the competitive model (severe winter temperatures) but 16 times better than the 2007 drought model (Table 3). The competitive model indicated that there were higher colony densities when the winter before the fall flight was less severe (coefficient = −0.0009; t = −2.7, df = 30, P = 0.011; adjusted R^2^ = 0.689).

**Table 3:** Weather models for beaver colony density on streams where no targeted beaver management occurred on the Chequamegon side of the Chequamegon-Nicolet National Forest, 1997–2013. Models listed are the minimum AIC model, competitive models (those models within 2 AC units of the 006Dinimum), and the 2007 drought model. The evidence ratio is the ratio of the Akaike weights for the minimum AIC model and an alternate model.

Models	n	K	Δ AIC_c_	Akaike weight	Evidence ratio
Trout stream type + Indicator of severe drought in 2007 + Deviance of mean maximum May temperature three years before fall flight	34	5	0	0.433	
Trout stream type + Indicator of severe drought in 2007 + Deviance of the number of December–March days below −17.8°C the year of fall flight	34	5	1.09	0.252	1.59
Trout stream type + Indicator of severe drought in 2007	34	3	5.55	0.023	16.06

On the Nicolet side, when we added the weather variables to the time trend model for non-targeted streams, there was a significant non-linear relationship with May PDSI three years prior to the fall flight (s(May PDSI lag 3): F = 3.14, df = 2.3, 45.35, P = 0.037; adjusted R^2^ = 0.896) (Table 4). Specifically, years that were in moderate drought or, conversely, moderately moist or wetter in the years before the 2-year-olds were born were associated with years with lower colony densities when those 2-year-olds dispersed. Using a baseline year of 1990 (to control for year), average predicted colony density when May PDSI values were in the moderate drought category (−2 to −2.99) was 0.196 colonies/km (standard error = 0.0009, n = 7), 0.209 colonies/km (standard error = 0.0015, n = 10) for the moderately moist category (2 to 2.99), and 0.222 colonies/km (standard error = 0.0005, n = 11) for mid-range values (−0.5 to 0.5) There were two competitive models. The first competitive model included August–October PDSI three years prior to the fall flight (i.e., food caching period before the females become pregnant) (s(Aug–Oct PDSI lag 3): F = 2.77, df = 1.9, 42.7, P = 0.064; adjusted R^2^ = 0.896) (Table 4). Under this model, moderate-severe drought in the fall before the female became pregnant was associated with lower colony densities when those 2-year-olds dispersed. Using a baseline year of 1990 (to control for year), average predicted colony density when August–October PDSI values were in the moderate-severe drought category (−2.5 to −3.3) was 0.197 colonies/km (standard error = 0.0008, n = 8), 0.222 colonies/km (standard error = 0.0006, n = 11) for mid-range values (−0.5 to 0.5), and 0.222 colonies/km (standard error = 0.0004, n = 9) for the moderately-very moist category (2.5 to 3.4). The second competitive model indicated that there were lower colony densities when the winter was more severe two years before the fall flight (i.e., winter the female became pregnant with the 2-year-old cohort) (coefficient = −0.0005; t = −2.13, df = 43.5, P = 0.039; adjusted R^2^ = 0.891).

**Table 4:** Time trend and weather models for beaver colony density on streams where no targeted beaver management occurred on the Nicolet side of the Chequamegon-Nicolet National Forest, 1987–2013. Models listed are the minimum AIC model, competitive models (those models within 2 AC units of the minimum), and the trend model.

Models	n	K	Δ AIC_c_	Akaike weight	Evidence ratio
s(Year) + s(May PDSI three years prior to fall flight)	54	11.9	0	0.256	
s(Year) + s(August–October PDSI three years prior to fall flight)	54	12.3	0.59	0.191	1.34
s(Year) + Standardized number of December–March days below -17.8°C two years before fall flight	54	11.5	1.48	0.122	2.09
s(Year)	54	10.2	4.54	0.032	9.68

The notation s() indicates a nonparametric smooth. K is the number of model parameters and the evidence ratio is the ratio of the Akaike weights for the minimum AIC model and an alternate model.

## Discussion

After 20 years of beaver control, we found a substantial and sustained Forest-wide reduction in beaver colony density on targeted trout streams across the Chequamegon-Nicolet National Forest. The magnitude of the reduction on both sides of the Forest was similar at roughly 60% relative to non-targeted trout streams—the targeted streams were regularly recolonized, but well below pre-control levels. Using the Chequamegon side as the benchmark, it took seven years of effort to achieve the full reduction, which was then sustained over the remainder of the monitoring period. The percentage declines are similar to the declines (but not the numerical harvest levels) documented in beaver pelt harvest records of the mid-1800s when commercial beaver trapping was occurring [34].

The Forest conducted a test in the early 1990s using only regulated trapping on a subset of its trout streams. This approach was not able to meet beaver reduction goals, and the test streams were reassigned to the targeted trapping program. It is an interesting question why regulated trapping was not able to generate a reduction similar to that found in the targeted beaver population. We speculate there was interplay between effort and population dynamics. Absent very high pelt prices, regulated trappers might have skipped pursuing beaver colonies that were located on harder-to-access reaches of the managed streams. This could have resulted in persistent colonies producing dispersing young that could colonize the parts of the streams that were accessible by trappers, keeping the annual fall flight population index data near unmanaged levels. USDA-WS trappers, on the other hand, are paid to get into harder-to-access areas and so might more effectively reduce the targeted beaver population.

While our analysis was based on a population index rather than a population estimate, our finding of higher colony densities on the Chequamegon side compared to the Nicolet side of the Forest is consistent with beaver population estimates for northern Wisconsin. Historically, after the over-trapping era of the late 1890s, remnant beaver populations remained in the far northwestern part of the state [35]. Currently, the beaver population in northwestern Wisconsin (location of the Chequamegon) is consistently higher than that for northeastern Wisconsin (location of the Nicolet) [36, 37]. Our finding of different time trends between the two sides of the Forest for colonies on non-targeted streams is also consistent with the time trends seen in the beaver population in northern Wisconsin. While the beaver population in northern Wisconsin declined overall between 1992 and 2008, the decline was steepest in northeastern Wisconsin and a sharp decline occurred between 1995 and 1998 [36]. The 1998 population estimate in northeastern Wisconsin declined by 47% from the 1995 estimate while the population in northwestern Wisconsin declined only 13% (calculated from [36]). The influence of the 2007 severe drought that we detected on the Chequamegon was likely a contributing factor to the 32% decline in the northwestern Wisconsin beaver population between 2005 and 2008, the steepest decline seen in the northwest beaver population (calculated from [36]).

Environmental conditions can be important factors causing mortality in beaver populations [18]. While our evaluation of weather influences was exploratory, the weather signals we found correspond to factors found in other studies that affect the size of the cohort of 2-year-old dispersers, either through a linkage with survival while in the den or to cohort size via female condition [21, 22]. In our study, though the specific weather variables associated with beaver colony density on the two sides of the Forest were different, the commonality was that dry conditions during spring and severe winters were associated with reduced beaver colony density in the fall (either the same year or lagged). Drought was implicated in the depression of local beaver populations in western Nebraska [18] and has been suggested as a cause of mortality in beaver in Kansas [38]. Severe winters and starvation have also been noted as principal causes of mortality in northern climates [6].

Beaver populations without extensive trapping can have complex dynamics including declines [39] and nonlinear trends [40]. The pattern we found on the Chequamegon side of the Forest (decline on the targeted trout streams and no pattern on the non-targeted streams) was the expected pattern if regulated trapping was at sustainable levels and randomly distributed across the streamscape, making targeted trapping the differentiating factor affecting colony density on trout streams. We did not find that pattern on the Nicolet side of the Forest. Instead we found substantial declines on the non-targeted streams as well. On the Nicolet, the observed 65% decline in the colony index from 1994–2013 could be attributable to either a set of local factors driving the population down apart from the targeted beaver management program (e.g., local concentrations of regulated trapping, changes in habitat-based carrying capacity) or it could be that targeted streams in the broader landscape had previously provided a source of colonizing beavers for the Forest (i.e., source-sink dynamics [39]). In northeast Wisconsin counties, 24% to 67% of total perennial stream kilometers are under targeted beaver management [1]. In contrast, in northwest Wisconsin, only in one county does targeted beaver management exceed 25% of perennial stream kilometers with most other counties having <10% of stream kilometers under targeted beaver management (Figure 10 in [1]). While the interaction between streams within the Forest and the broader landscape is not known, we do know that new beaver colonies on the targeted streams in the Forest arise only through new colonization each year and these colonies are removed the following spring, before they can produce dispersing young. Currently, even with declining populations, the Nicolet non-targeted streams are providing colonizers for the targeted streams at the same relative rate as occurs in the Chequamegon system (generating a targeted colony density 60% lower than the non-targeted colony density in both the Chequamegon and Nicolet systems).

Many public land management organizations are faced with optimizing and prioritizing resource allocations to competing management objectives. The question is whether the desired cold-water ecosystem and trout benefits can be maintained if the intensity of targeted beaver management, and thus its cost, is reduced. Our finding that colony densities vary temporally and spatially may help refine management options across the Forest and the state to lower the cost of beaver management. Relying more heavily on targeting densities by region, watersheds, specific locations, or by stream systems may be a means of transitioning to variable trapping pressures over larger regions. For example, stable cold-water groundwater inputs or extensive groundwater inputs combined with vegetative stream shading were able to maintain downstream water temperatures in northern Wisconsin despite the presence of beaver dams [10]. Thus, “judicious removal of a few key beaver impoundments may have a strong effect on downstream temperatures without the loss of diversity associated with large-scale dam removal” [10]. Management programs using variable trapping can also integrate other beaver damage factors beyond fisheries such as infrastructure and bank stabilization. While understanding local impacts of beavers on trout fisheries is an important research topic in the state of Wisconsin [1], further investigation on how local stream characteristics plus surrounding landscapes influence beaver recolonization events and territory settlement patterns will be important to help land management agencies refine large-scale beaver management programs.
